# Supplementary material for: Genetic architecture of inter-specific and -generic grass hybrids by network analysis on multi-omics data
Source: BMC Genomics. 2023 Apr 25;24:213. doi: 10.1186/s12864-023-09292-7 (PMC10127077; doi:10.1186/s12864-023-09292-7)
Supplement: Supplementary file 1 — Additional file 1. Genetic structure via principal component analysis. [file 12864_2023_9292_MOESM1_ESM.pdf]

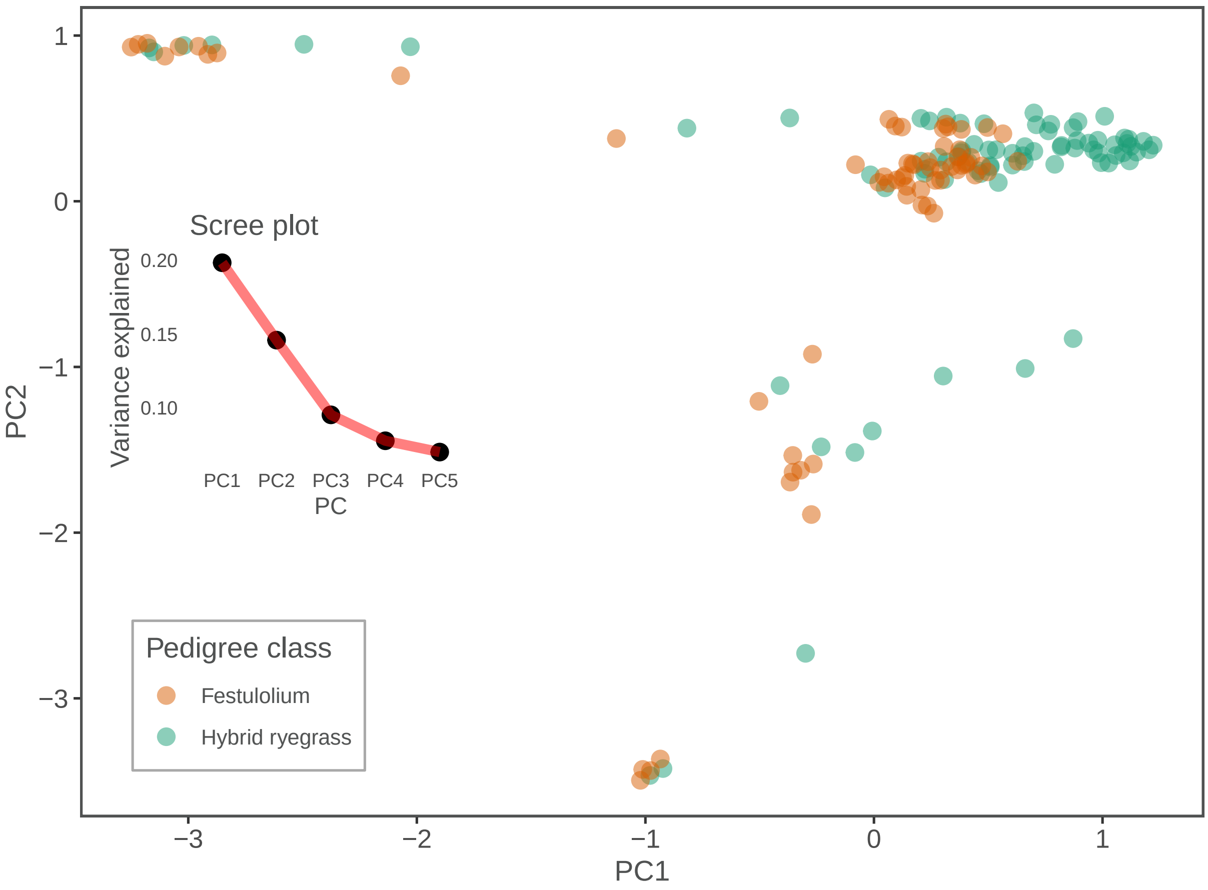

Figure S1: Genetic structure via principal component analysis. The scatter plot displays scores of the first two principal components (PCs) from the PC analysis of the combined (hybrid ryegrass plus *Festulolium loliaceum* samples) genomic relationship matrix. The number of samples is equal to 144. An overlaying scree plot shows the variance explained by the first five PCs.
