# Supplementary material for: Genetic architecture of inter-specific and -generic grass hybrids by network analysis on multi-omics data
Source: BMC Genomics. 2023 Apr 25;24:213. doi: 10.1186/s12864-023-09292-7 (PMC10127077; doi:10.1186/s12864-023-09292-7)
Supplement: Supplementary file 2 — Additional file 2. Variance components estimated via Restricted maximum likelihood (REML). [file 12864_2023_9292_MOESM2_ESM.pdf]

Table S1: Variance components estimated via Restricted maximum likelihood (REML). Estimates of variance components were estimated independently for two field trials comprising different pedigree classes of forage grasses: HR - hybrid ryegrass and FL - *Festulolium loliaceum*.

Ped. class	Variance component	Traits[1]							
		DMY	ADF	ADL	DMDig	NDF	NDFD	Prot	WSC
HR	σ_u^2	2181.088	0.125	0.002	0.135	0.278	1.451	0.186	0.000
	S.E.	1054.606	0.073	0.002	0.086	0.174	0.741	0.113	0.368
FL	σ_u^2	7353.689	0.260	0.018	0.148	0.590	0.000	0.034	0.194
	S.E.	3090.586	0.128	0.011	0.281	0.277	0.540	0.046	0.162
HR	σ_f^2	0.000	0.000	0.000	0.031	0.029	0.000	0.000	0.269
	S.E.	892.753	0.075	0.002	0.099	0.183	0.859	0.117	0.587
FL	σ_f^2	0.000	0.000	0.000	0.374	0.102	0.704	0.071	0.000
	S.E.	1760.040	0.099	0.008	0.441	0.186	0.976	0.066	0.200
HR	σ_s^2	183.365	0.206	0.007	0.222	0.527	0.253	0.377	1.530
	S.E.	721.615	0.106	0.003	0.136	0.263	0.547	0.193	0.810
FL	σ_s^2	4431.405	0.443	0.000	0.000	0.402	0.228	0.039	1.835
	S.E.	2338.886	0.194	0.008	0.443	0.254	0.927	0.056	0.599
HR	σ_e^2	4821.160	0.388	0.010	0.512	0.925	4.189	0.561	3.242
	S.E.	986.994	0.085	0.002	0.115	0.210	0.722	0.139	0.740
FL	σ_e^2	5939.760	0.361	0.046	2.400	0.728	5.799	0.319	0.659
	S.E.	1618.810	0.114	0.010	0.528	0.202	1.225	0.073	0.286
HR	μ	1312.640	23.420	2.110	88.937	42.872	71.172	14.143	10.986
	S.E.	20.718	0.301	0.051	0.326	0.477	0.626	0.391	0.824
FL	μ	1204.610	26.757	2.912	85.493	46.501	70.291	11.720	12.422
	S.E.	50.153	0.442	0.084	0.603	0.517	0.942	0.255	0.782
HR	h^2	0.304	0.174	0.126	0.150	0.158	0.246	0.165	0.000
FL	h^2	0.415	0.244	0.288	0.051	0.324	0.000	0.074	0.072

[1]DMY: dry matter yield; ADF: acid detergent fiber; ADL: acid detergent lignin; DMDig: digestible dry matter; NDF: neutral detergent fiber; NDFD: digestible NDF; Prot: protein; and WSC: water-soluble carbohydrate. σ_u^2, σ_f^2, σ_s^2, and σ_e^2 are the genomic variance, variance due to uncorrelated family effects, spatial variance, and residual variance, respectively. S.E. is the asymptotic standard error and h^2 is the genomic heritability.
