# Supplementary material for: Genetic architecture of inter-specific and -generic grass hybrids by network analysis on multi-omics data
Source: BMC Genomics. 2023 Apr 25;24:213. doi: 10.1186/s12864-023-09292-7 (PMC10127077; doi:10.1186/s12864-023-09292-7)
Supplement: Supplementary file 3 — Additional file 3. Exploring the genomic and NMR landscape of hub nodes. [file 12864_2023_9292_MOESM3_ESM.pdf]

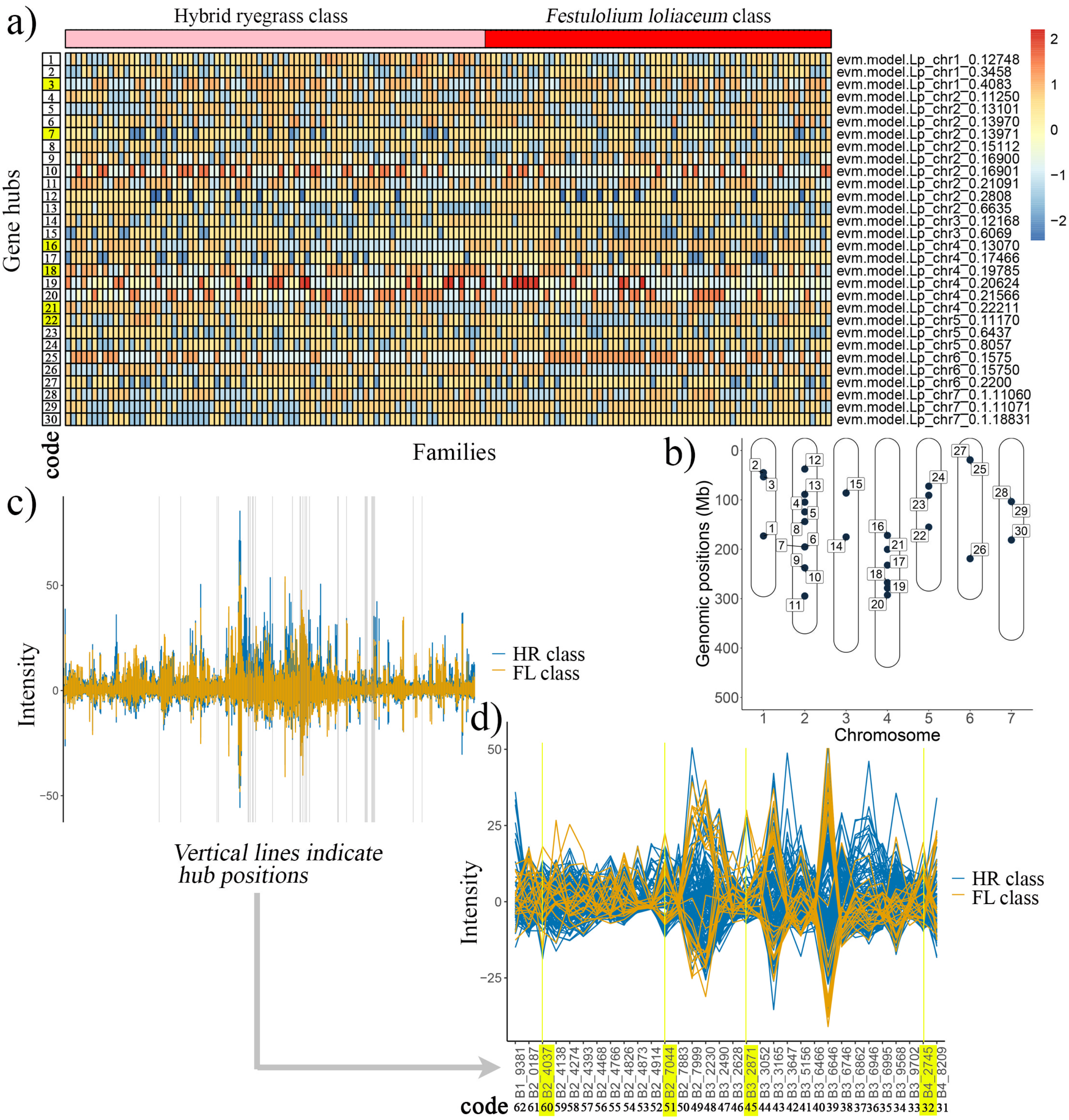

Figure S2: Hub nodes are well distributed throughout the genome and NMR spectrum. Panel a shows a heatmap of log10-transformed expression data for the 30 hub genes, with hub codes listed on the left of the plot. In panel b, the genomic locations of the hub genes are plotted across all seven chromosomes. Panel c depicts Pareto-transformed, bucketed NMR intensities across the entire spectrum, with vertical lines indicating hub positions. Finally, in panel d, the intensities of the 32 hubs are displayed, with hub codes listed at the bottom of the plot. Hub genes and unidentified NMR compounds that have at least one significant edge with phenotypes (see Figure 5) are highlighted in yellow.
